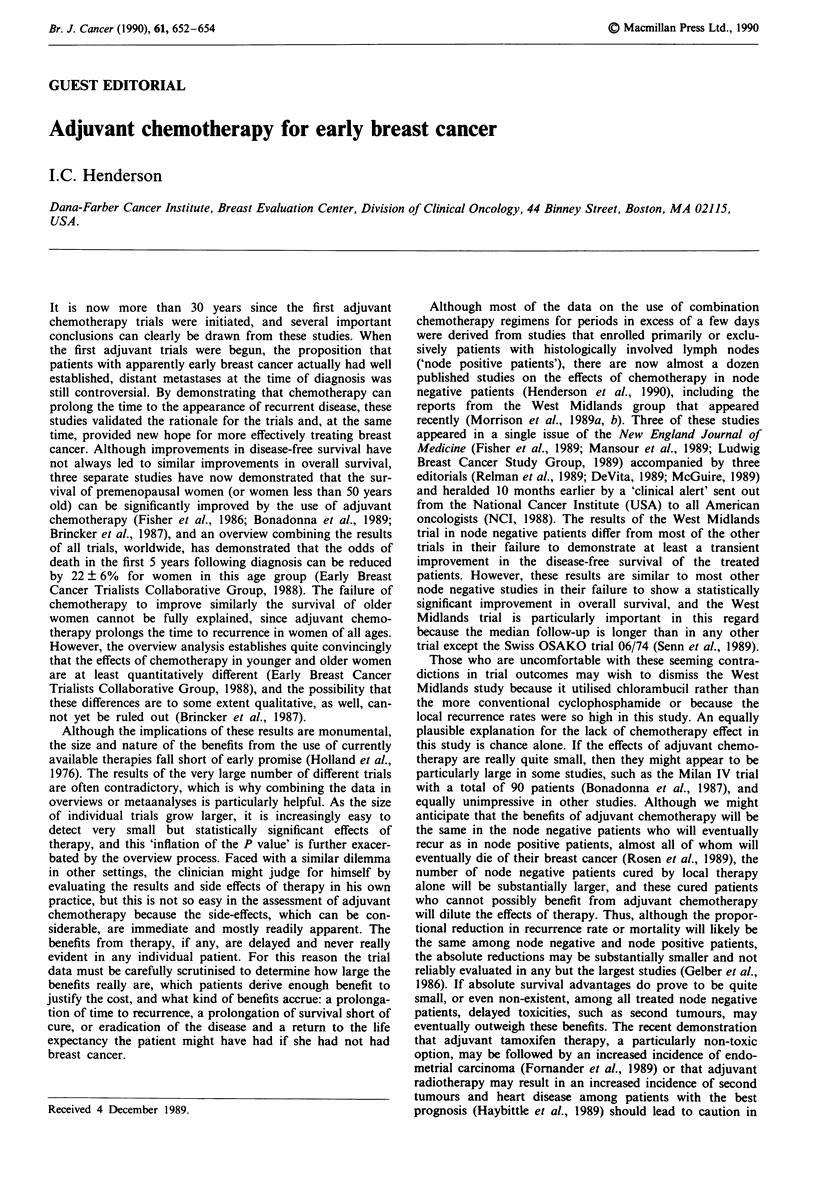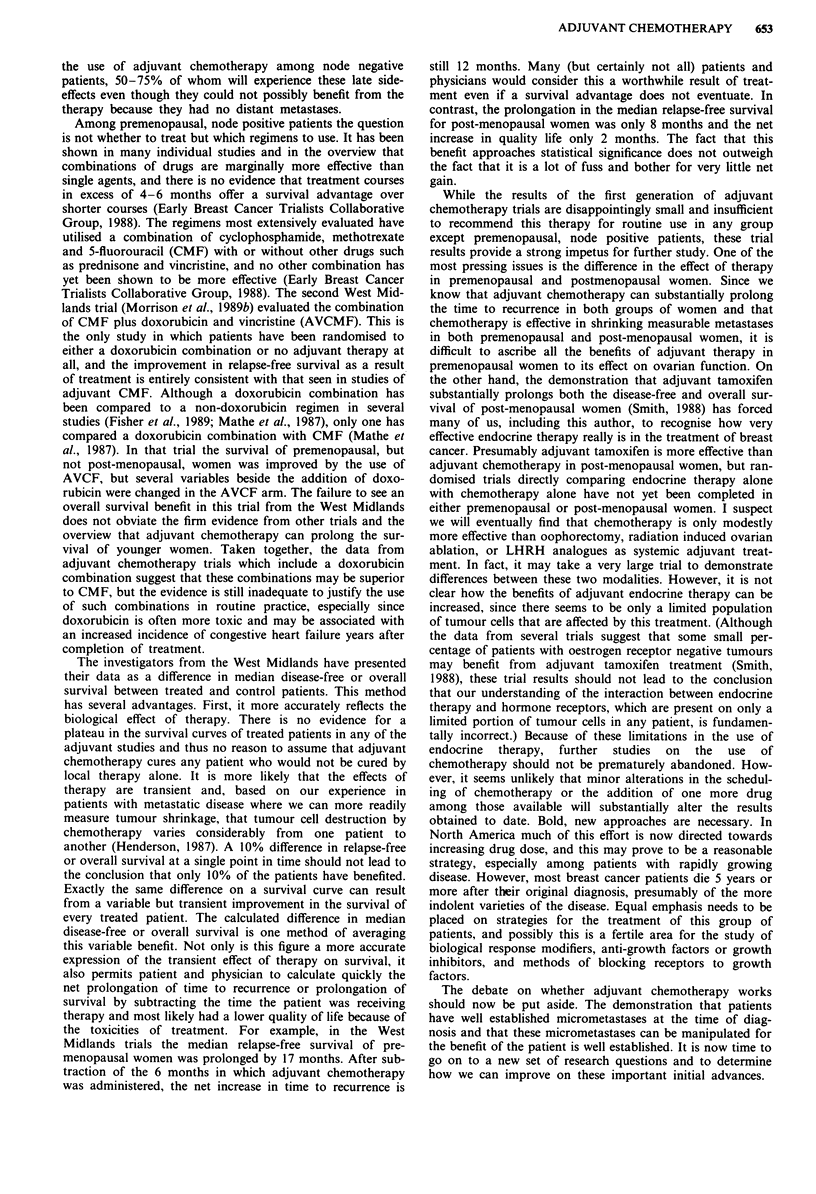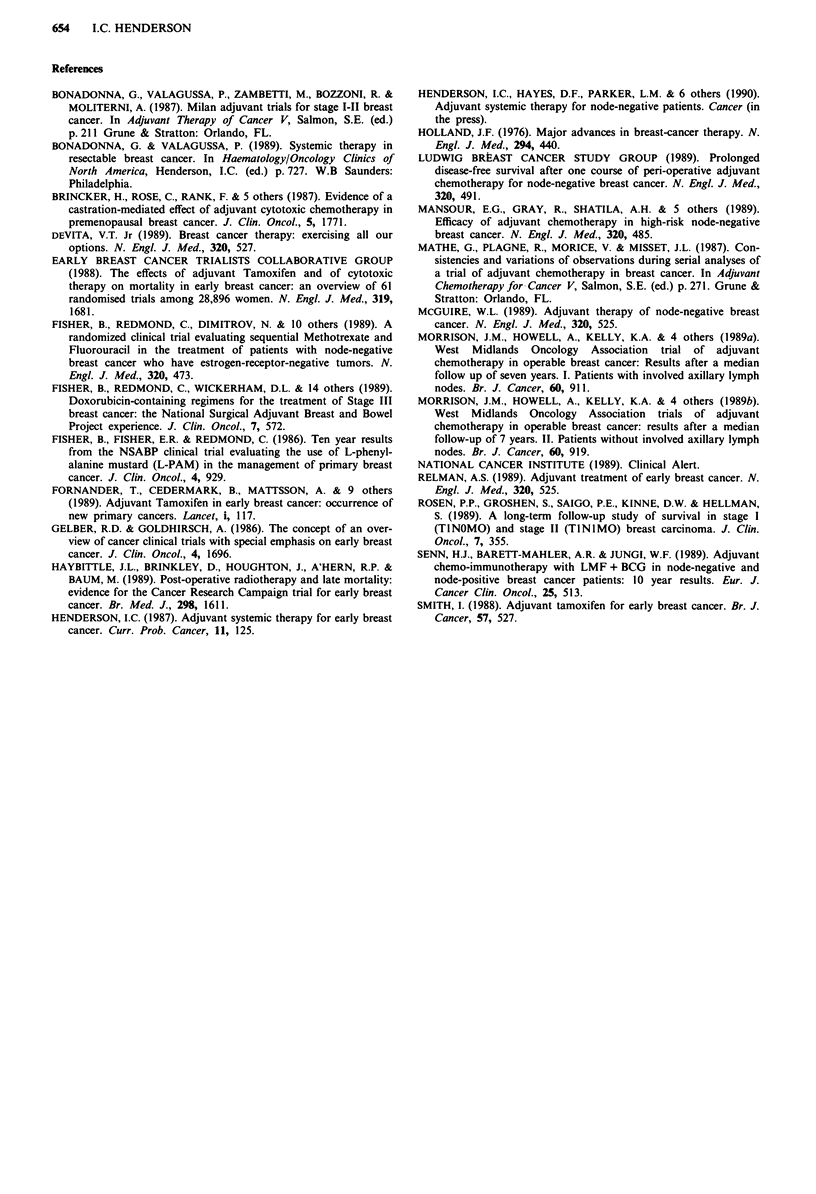# Adjuvant chemotherapy for early breast cancer.

**DOI:** 10.1038/bjc.1990.147

**Published:** 1990-05

**Authors:** I. C. Henderson

**Affiliations:** Dana-Farber Cancer Institute, Breast Evaluation Center, Division of Clinical Oncology, Boston, MA 02115.


Br. J. Cancer (1990), 61, 652-654 © Macmillan Press Ltd., 1990

GUEST EDITORIAL

### Adjuvant chemotherapy for early breast cancer

I.C. Henderson

*Dana-Farber Cancer Institute, Breast Evaluation Center, Division of Clinical Oncology, 44 Binney Street, Boston, MA 02115, USA.*

It is now more than 30 years since the first adjuvant chemotherapy trials were initiated, and several important conclusions can clearly be drawn from these studies. When the first adjuvant trials were begun, the proposition that patients with apparently early breast cancer actually had well established, distant metastases at the time of diagnosis was still controversial. By demonstrating that chemotherapy can prolong the time to the appearance of recurrent disease, these studies validated the rationale for the trials and, at the same time, provided new hope for more effectively treating breast cancer. Although improvements in disease-free survival have not always led to similar improvements in overall survival, three separate studies have now demonstrated that the survival of premenopausal women (or women less than 50 years old) can be significantly improved by the use of adjuvant chemotherapy (Fisher *et al.*, 1986; Bonadonna *et al.*, 1989; Brincker *et al.*, 1987), and an overview combining the results of all trials, worldwide, has demonstrated that the odds of death in the first 5 years following diagnosis can be reduced by $22 \pm 6\%$ for women in this age group (Early Breast Cancer Trialists Collaborative Group, 1988). The failure of chemotherapy to improve similarly the survival of older women cannot be fully explained, since adjuvant chemotherapy prolongs the time to recurrence in women of all ages. However, the overview analysis establishes quite convincingly that the effects of chemotherapy in younger and older women are at least quantitatively different (Early Breast Cancer Trialists Collaborative Group, 1988), and the possibility that these differences are to some extent qualitative, as well, cannot yet be ruled out (Brincker *et al.*, 1987).

Although the implications of these results are monumental, the size and nature of the benefits from the use of currently available therapies fall short of early promise (Holland *et al.*, 1976). The results of the very large number of different trials are often contradictory, which is why combining the data in overviews or metaanalyses is particularly helpful. As the size of individual trials grow larger, it is increasingly easy to detect very small but statistically significant effects of therapy, and this 'inflation of the *P* value' is further exacerbated by the overview process. Faced with a similar dilemma in other settings, the clinician might judge for himself by evaluating the results and side effects of therapy in his own practice, but this is not so easy in the assessment of adjuvant chemotherapy because the side-effects, which can be considerable, are immediate and mostly readily apparent. The benefits from therapy, if any, are delayed and never really evident in any individual patient. For this reason the trial data must be carefully scrutinised to determine how large the benefits really are, which patients derive enough benefit to justify the cost, and what kind of benefits accrue: a prolongation of time to recurrence, a prolongation of survival short of cure, or eradication of the disease and a return to the life expectancy the patient might have had if she had not had breast cancer.

Received 4 December 1989.

Although most of the data on the use of combination chemotherapy regimens for periods in excess of a few days were derived from studies that enrolled primarily or exclusively patients with histologically involved lymph nodes ('node positive patients'), there are now almost a dozen published studies on the effects of chemotherapy in node negative patients (Henderson *et al.*, 1990), including the reports from the West Midlands group that appeared recently (Morrison *et al.*, 1989*a*, *b*). Three of these studies appeared in a single issue of the *New England Journal of Medicine* (Fisher *et al.*, 1989; Mansour *et al.*, 1989; Ludwig Breast Cancer Study Group, 1989) accompanied by three editorials (Relman *et al.*, 1989; DeVita, 1989; McGuire, 1989) and heralded 10 months earlier by a 'clinical alert' sent out from the National Cancer Institute (USA) to all American oncologists (NCI, 1988). The results of the West Midlands trial in node negative patients differ from most of the other trials in their failure to demonstrate at least a transient improvement in the disease-free survival of the treated patients. However, these results are similar to most other node negative studies in their failure to show a statistically significant improvement in overall survival, and the West Midlands trial is particularly important in this regard because the median follow-up is longer than in any other trial except the Swiss OSAKO trial 06/74 (Senn *et al.*, 1989).

Those who are uncomfortable with these seeming contradictions in trial outcomes may wish to dismiss the West Midlands study because it utilised chlorambucil rather than the more conventional cyclophosphamide or because the local recurrence rates were so high in this study. An equally plausible explanation for the lack of chemotherapy effect in this study is chance alone. If the effects of adjuvant chemotherapy are really quite small, then they might appear to be particularly large in some studies, such as the Milan IV trial with a total of 90 patients (Bonadonna *et al.*, 1987), and equally unimpressive in other studies. Although we might anticipate that the benefits of adjuvant chemotherapy will be the same in the node negative patients who will eventually recur as in node positive patients, almost all of whom will eventually die of their breast cancer (Rosen *et al.*, 1989), the number of node negative patients cured by local therapy alone will be substantially larger, and these cured patients who cannot possibly benefit from adjuvant chemotherapy will dilute the effects of therapy. Thus, although the proportional reduction in recurrence rate or mortality will likely be the same among node negative and node positive patients, the absolute reductions may be substantially smaller and not reliably evaluated in any but the largest studies (Gelber *et al.*, 1986). If absolute survival advantages do prove to be quite small, or even non-existent, among all treated node negative patients, delayed toxicities, such as second tumours, may eventually outweigh these benefits. The recent demonstration that adjuvant tamoxifen therapy, a particularly non-toxic option, may be followed by an increased incidence of endometrial carcinoma (Fornander *et al.*, 1989) or that adjuvant radiotherapy may result in an increased incidence of second tumours and heart disease among patients with the best prognosis (Haybittle *et al.*, 1989) should lead to caution in the use of adjuvant chemotherapy among node negative patients, 50–75% of whom will experience these late side-effects even though they could not possibly benefit from the therapy because they had no distant metastases.

Among premenopausal, node positive patients the question is not whether to treat but which regimens to use. It has been shown in many individual studies and in the overview that combinations of drugs are marginally more effective than single agents, and there is no evidence that treatment courses in excess of 4–6 months offer a survival advantage over shorter courses (Early Breast Cancer Trialists Collaborative Group, 1988). The regimens most extensively evaluated have utilised a combination of cyclophosphamide, methotrexate and 5-fluorouracil (CMF) with or without other drugs such as prednisone and vincristine, and no other combination has yet been shown to be more effective (Early Breast Cancer Trialists Collaborative Group, 1988). The second West Midlands trial (Morrison *et al.*, 1989*b*) evaluated the combination of CMF plus doxorubicin and vincristine (AVCMF). This is the only study in which patients have been randomised to either a doxorubicin combination or no adjuvant therapy at all, and the improvement in relapse-free survival as a result of treatment is entirely consistent with that seen in studies of adjuvant CMF. Although a doxorubicin combination has been compared to a non-doxorubicin regimen in several studies (Fisher *et al.*, 1989; Mathe *et al.*, 1987), only one has compared a doxorubicin combination with CMF (Mathe *et al.*, 1987). In that trial the survival of premenopausal, but not post-menopausal, women was improved by the use of AVCF, but several variables beside the addition of doxorubicin were changed in the AVCF arm. The failure to see an overall survival benefit in this trial from the West Midlands does not obviate the firm evidence from other trials and the overview that adjuvant chemotherapy can prolong the survival of younger women. Taken together, the data from adjuvant chemotherapy trials which include a doxorubicin combination suggest that these combinations may be superior to CMF, but the evidence is still inadequate to justify the use of such combinations in routine practice, especially since doxorubicin is often more toxic and may be associated with an increased incidence of congestive heart failure years after completion of treatment.

The investigators from the West Midlands have presented their data as a difference in median disease-free or overall survival between treated and control patients. This method has several advantages. First, it more accurately reflects the biological effect of therapy. There is no evidence for a plateau in the survival curves of treated patients in any of the adjuvant studies and thus no reason to assume that adjuvant chemotherapy cures any patient who would not be cured by local therapy alone. It is more likely that the effects of therapy are transient and, based on our experience in patients with metastatic disease where we can more readily measure tumour shrinkage, that tumour cell destruction by chemotherapy varies considerably from one patient to another (Henderson, 1987). A 10% difference in relapse-free or overall survival at a single point in time should not lead to the conclusion that only 10% of the patients have benefited. Exactly the same difference on a survival curve can result from a variable but transient improvement in the survival of every treated patient. The calculated difference in median disease-free or overall survival is one method of averaging this variable benefit. Not only is this figure a more accurate expression of the transient effect of therapy on survival, it also permits patient and physician to calculate quickly the net prolongation of time to recurrence or prolongation of survival by subtracting the time the patient was receiving therapy and most likely had a lower quality of life because of the toxicities of treatment. For example, in the West Midlands trials the median relapse-free survival of premenopausal women was prolonged by 17 months. After subtraction of the 6 months in which adjuvant chemotherapy was administered, the net increase in time to recurrence is still 12 months. Many (but certainly not all) patients and physicians would consider this a worthwhile result of treatment even if a survival advantage does not eventuate. In contrast, the prolongation in the median relapse-free survival for post-menopausal women was only 8 months and the net increase in quality life only 2 months. The fact that this benefit approaches statistical significance does not outweigh the fact that it is a lot of fuss and bother for very little net gain.

While the results of the first generation of adjuvant chemotherapy trials are disappointingly small and insufficient to recommend this therapy for routine use in any group except premenopausal, node positive patients, these trial results provide a strong impetus for further study. One of the most pressing issues is the difference in the effect of therapy in premenopausal and postmenopausal women. Since we know that adjuvant chemotherapy can substantially prolong the time to recurrence in both groups of women and that chemotherapy is effective in shrinking measurable metastases in both premenopausal and post-menopausal women, it is difficult to ascribe all the benefits of adjuvant therapy in premenopausal women to its effect on ovarian function. On the other hand, the demonstration that adjuvant tamoxifen substantially prolongs both the disease-free and overall survival of post-menopausal women (Smith, 1988) has forced many of us, including this author, to recognise how very effective endocrine therapy really is in the treatment of breast cancer. Presumably adjuvant tamoxifen is more effective than adjuvant chemotherapy in post-menopausal women, but randomised trials directly comparing endocrine therapy alone with chemotherapy alone have not yet been completed in either premenopausal or post-menopausal women. I suspect we will eventually find that chemotherapy is only modestly more effective than oophorectomy, radiation induced ovarian ablation, or LHRH analogues as systemic adjuvant treatment. In fact, it may take a very large trial to demonstrate differences between these two modalities. However, it is not clear how the benefits of adjuvant endocrine therapy can be increased, since there seems to be only a limited population of tumour cells that are affected by this treatment. (Although the data from several trials suggest that some small percentage of patients with oestrogen receptor negative tumours may benefit from adjuvant tamoxifen treatment (Smith, 1988), these trial results should not lead to the conclusion that our understanding of the interaction between endocrine therapy and hormone receptors, which are present on only a limited portion of tumour cells in any patient, is fundamentally incorrect.) Because of these limitations in the use of endocrine therapy, further studies on the use of chemotherapy should not be prematurely abandoned. However, it seems unlikely that minor alterations in the scheduling of chemotherapy or the addition of one more drug among those available will substantially alter the results obtained to date. Bold, new approaches are necessary. In North America much of this effort is now directed towards increasing drug dose, and this may prove to be a reasonable strategy, especially among patients with rapidly growing disease. However, most breast cancer patients die 5 years or more after their original diagnosis, presumably of the more indolent varieties of the disease. Equal emphasis needs to be placed on strategies for the treatment of this group of patients, and possibly this is a fertile area for the study of biological response modifiers, anti-growth factors or growth inhibitors, and methods of blocking receptors to growth factors.

The debate on whether adjuvant chemotherapy works should now be put aside. The demonstration that patients have well established micrometastases at the time of diagnosis and that these micrometastases can be manipulated for the benefit of the patient is well established. It is now time to go on to a new set of research questions and to determine how we can improve on these important initial advances.

### References

BONADONNA, G., VALAGUSSA, P., ZAMBETTI, M., BOZZONI, R. & MOLITERNI, A. (1987). Milan adjuvant trials for stage I-II breast cancer. In *Adjuvant Therapy of Cancer V*, Salmon, S.E. (ed.) p. 211 Grune & Stratton: Orlando, FL.

BONADONNA, G. & VALAGUSSA, P. (1989). Systemic therapy in resectable breast cancer. In *Haematology/Oncology Clinics of North America*, Henderson, I.C. (ed.) p. 727. W.B Saunders: Philadelphia.

BRINCKER, H., ROSE, C., RANK, F. & 5 others (1987). Evidence of a castration-mediated effect of adjuvant cytotoxic chemotherapy in premenopausal breast cancer. *J. Clin. Oncol.*, **5**, 1771.

DEVITA, V.T. Jr (1989). Breast cancer therapy: exercising all our options. *N. Engl. J. Med.*, **320**, 527.

EARLY BREAST CANCER TRIALISTS COLLABORATIVE GROUP (1988). The effects of adjuvant Tamoxifen and of cytotoxic therapy on mortality in early breast cancer: an overview of 61 randomised trials among 28,896 women. *N. Engl. J. Med.*, **319**, 1681.

FISHER, B., REDMOND, C., DIMITROV, N. & 10 others (1989). A randomized clinical trial evaluating sequential Methotrexate and Fluorouracil in the treatment of patients with node-negative breast cancer who have estrogen-receptor-negative tumors. *N. Engl. J. Med.*, **320**, 473.

FISHER, B., REDMOND, C., WICKERHAM, D.L. & 14 others (1989). Doxorubicin-containing regimens for the treatment of Stage III breast cancer: the National Surgical Adjuvant Breast and Bowel Project experience. *J. Clin. Oncol.*, **7**, 572.

FISHER, B., FISHER, E.R. & REDMOND, C. (1986). Ten year results from the NSABP clinical trial evaluating the use of L-phenylalanine mustard (L-PAM) in the management of primary breast cancer. *J. Clin. Oncol.*, **4**, 929.

FORNANDER, T., CEDERMARK, B., MATTSSON, A. & 9 others (1989). Adjuvant Tamoxifen in early breast cancer: occurrence of new primary cancers. *Lancet*, **i**, 117.

GELBER, R.D. & GOLDHIRSCH, A. (1986). The concept of an overview of cancer clinical trials with special emphasis on early breast cancer. *J. Clin. Oncol.*, **4**, 1696.

HAYBITTLE, J.L., BRINKLEY, D., HOUGHTON, J., A'HERN, R.P. & BAUM, M. (1989). Post-operative radiotherapy and late mortality: evidence for the Cancer Research Campaign trial for early breast cancer. *Br. Med. J.*, **298**, 1611.

HENDERSON, I.C. (1987). Adjuvant systemic therapy for early breast cancer. *Curr. Prob. Cancer*, **11**, 125.

HENDERSON, I.C., HAYES, D.F., PARKER, L.M. & 6 others (1990). Adjuvant systemic therapy for node-negative patients. *Cancer* (in the press).

HOLLAND, J.F. (1976). Major advances in breast-cancer therapy. *N. Engl. J. Med.*, **294**, 440.

LUDWIG BREAST CANCER STUDY GROUP (1989). Prolonged disease-free survival after one course of peri-operative adjuvant chemotherapy for node-negative breast cancer. *N. Engl. J. Med.*, **320**, 491.

MANSOUR, E.G., GRAY, R., SHATILA, A.H. & 5 others (1989). Efficacy of adjuvant chemotherapy in high-risk node-negative breast cancer. *N. Engl. J. Med.*, **320**, 485.

MATHE, G., PLAGNE, R., MORICE, V. & MISSET, J.L. (1987). Consistencies and variations of observations during serial analyses of a trial of adjuvant chemotherapy in breast cancer. In *Adjuvant Chemotherapy for Cancer V*, Salmon, S.E. (ed.) p. 271. Grune & Stratton: Orlando, FL.

MCGUIRE, W.L. (1989). Adjuvant therapy of node-negative breast cancer. *N. Engl. J. Med.*, **320**, 525.

MORRISON, J.M., HOWELL, A., KELLY, K.A. & 4 others (1989*a*). West Midlands Oncology Association trial of adjuvant chemotherapy in operable breast cancer: Results after a median follow up of seven years. I. Patients with involved axillary lymph nodes. *Br. J. Cancer*, **60**, 911.

MORRISON, J.M., HOWELL, A., KELLY, K.A. & 4 others (1989*b*). West Midlands Oncology Association trials of adjuvant chemotherapy in operable breast cancer: results after a median follow-up of 7 years. II. Patients without involved axillary lymph nodes. *Br. J. Cancer*, **60**, 919.

NATIONAL CANCER INSTITUTE (1989). Clinical Alert.

RELMAN, A.S. (1989). Adjuvant treatment of early breast cancer. *N. Engl. J. Med.*, **320**, 525.

ROSEN, P.P., GROSHEN, S., SAIGO, P.E., KINNE, D.W. & HELLMAN, S. (1989). A long-term follow-up study of survival in stage I (T1N0M0) and stage II (T1N1M0) breast carcinoma. *J. Clin. Oncol.*, **7**, 355.

SENN, H.J., BARETT-MAHLER, A.R. & JUNGI, W.F. (1989). Adjuvant chemo-immunotherapy with LMF + BCG in node-negative and node-positive breast cancer patients: 10 year results. *Eur. J. Cancer Clin. Oncol.*, **25**, 513.

SMITH, I. (1988). Adjuvant tamoxifen for early breast cancer. *Br. J. Cancer*, **57**, 527.